# An interactive problem-solving approach to teach traumatology for medical students

**DOI:** 10.1186/1749-7922-5-24

**Published:** 2010-08-13

**Authors:** Fikri M Abu-Zidan, Margaret A Elzubeir

**Affiliations:** 1 Department of Surgery, Faculty of Medicine, United Arab Emirates University, UAE; 2 Department of Surgery, Faculty of Medicine, Auckland University, New Zealand; 3 College of Medicine, Department of Medical Education, King Saud bin Abdul Aziz University for Health Sciences, Riyadh, Saudi Arabia

## Abstract

**Aim:**

We aimed to evaluate an interactive problem-solving approach for teaching traumatology from perspectives of students and consider its implications on Faculty development.

**Methods:**

A two hour problem-solving, interactive tutorial on traumatology was structured to cover main topics in trauma management. The tutorial was based on real cases covering specific topics and objectives. Seven tutorials (5-9 students in each) were given by the same tutor with the same format for fourth and fifth year medical students in Auckland and UAE Universities (n = 50). A 16 item questionnaire, on a 7 point Likert-type scale, focusing on educational tools, tutor-based skills, and student-centered skills were answered by the students followed by open ended comments.

**Results:**

The tutorials were highly ranked by the students. The mean values of educational tools was the highest followed by tutor-centered skills and finally student-centered skills. There was a significant increase of the rating of studied attributes over time (F = 3.9, p = 0.004, ANOVA). Students' open ended comments were highly supportive of the interactive problem-solving approach for teaching traumatology.

**Conclusions:**

The interactive problem-solving approach for tutorials can be an effective enjoyable alternative or supplement to traditional instruction for teaching traumatology to medical students. Training for this approach should be encouraged for Faculty development.

## Introduction

Students often criticize lectures for limited opportunities for active involvement, interaction with the instructor, task-centered problem-solving opportunities, variation of activities and feedback on efforts [1,2]. The interactive approach for teaching however, involves an increased interchange between lecturer, students and the lecture content; promoting active involvement of students [3]. They are among innovative approaches for teaching and learning in medicine underpinned by adult learning principles [4] and are increasingly considered best educational practice that medical schools internationally are adopting as they revitalize their curriculum and shift to a learner-centered focus.

While this is important, it is equally imperative to seek students' input regarding quality of teaching and learning approaches experienced. The most often used evaluation tool is student ratings on different dimensions of the instructional process and presentation style [5]. We aimed to evaluate an interactive problem-solving approach for teaching traumatology from perspectives of students and consider its implications on Faculty development.

## Subjects and methods

### Educational material

**Figure 1:**
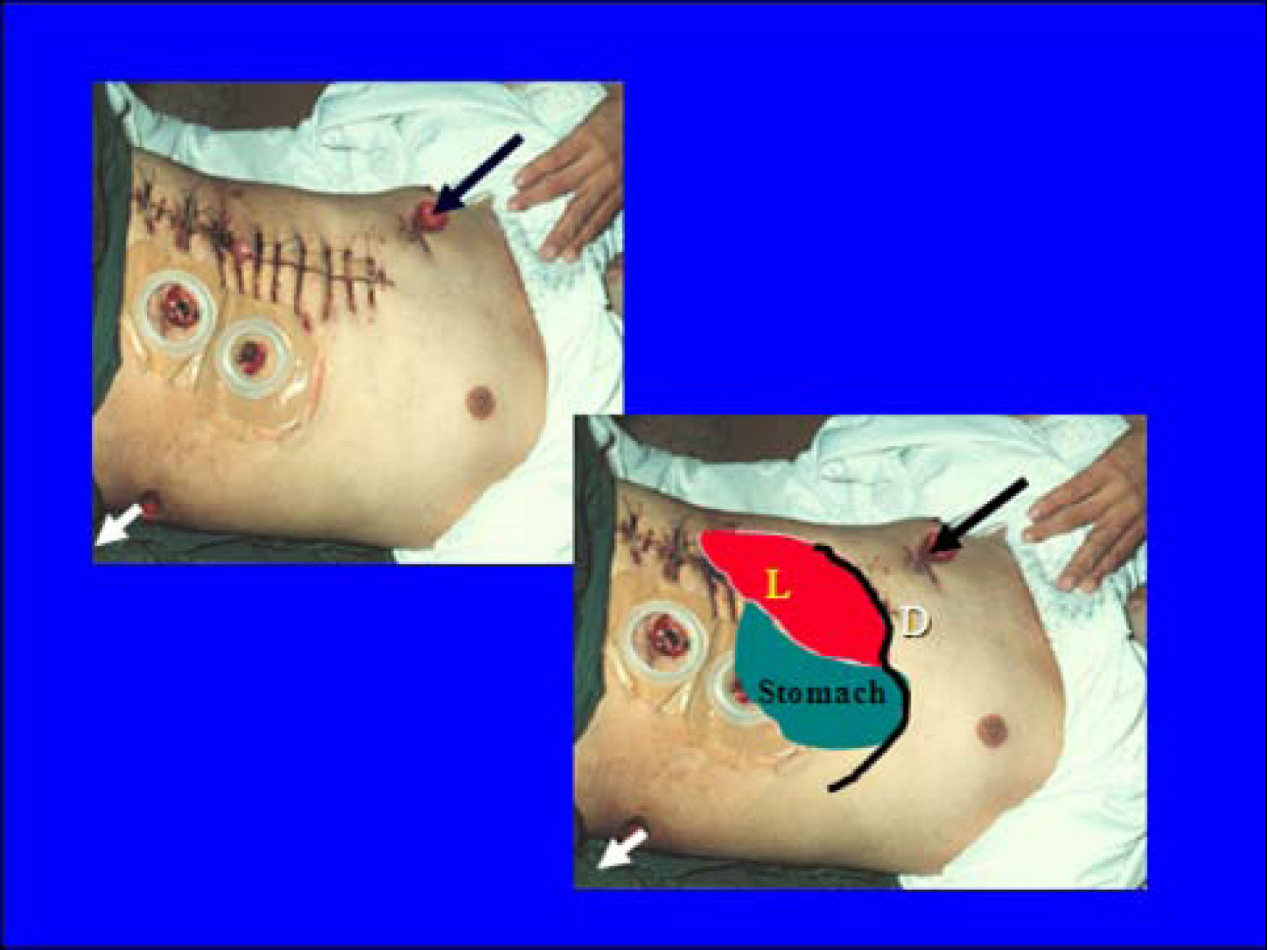
**A 20-years old patient who sustained a high energy bullet injury having an inlet at the right side of chest with an exit in the left loin**. L = liver, D = diaphragm, arrows show the inlet and exit of the bullet.

**Figure 2:**
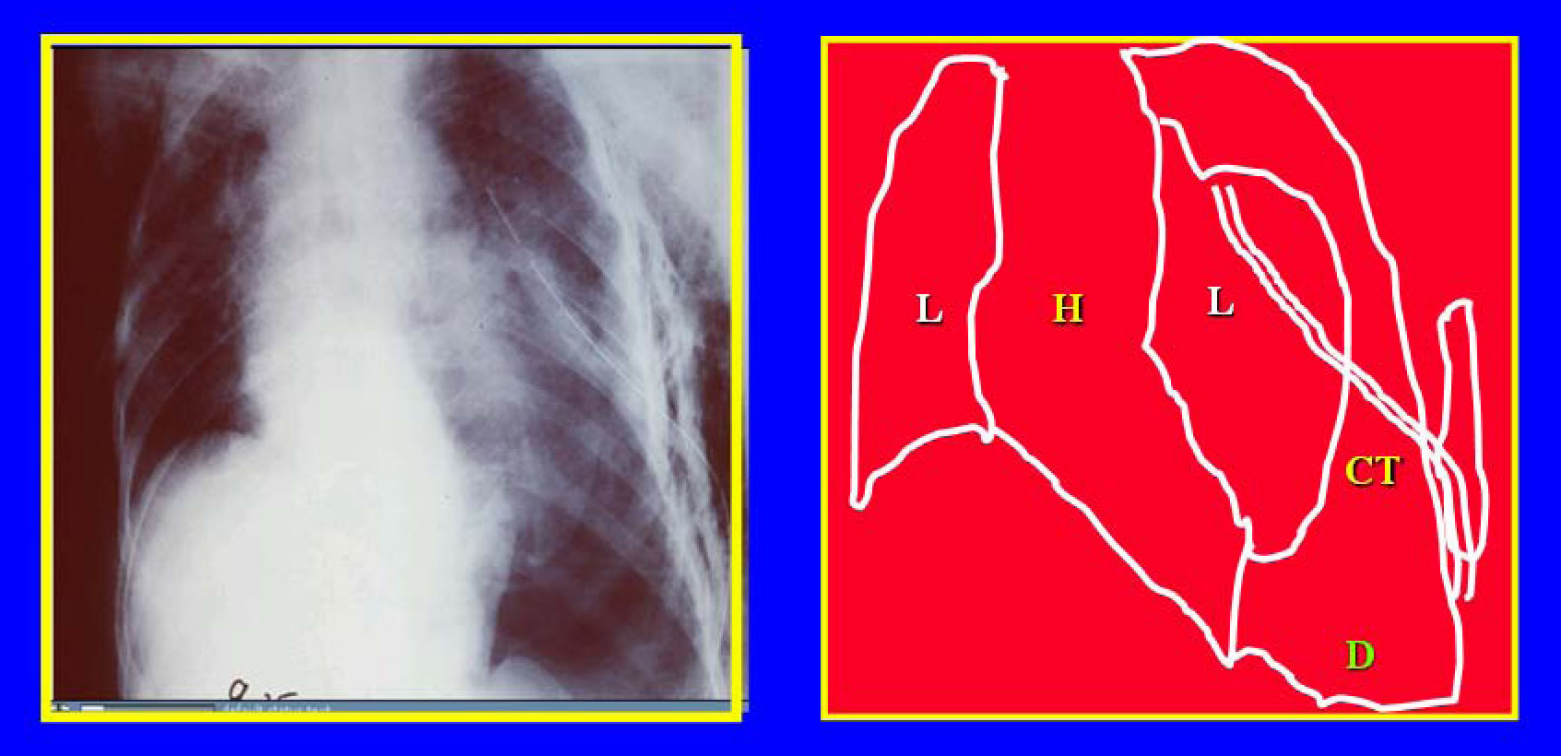
**A 45-years old male who developed severe hypoxia while being ventilated despite having a chest tube**. L = lung, H = heart, CT = chest tube, D = diaphragm.

**Figure 3:**
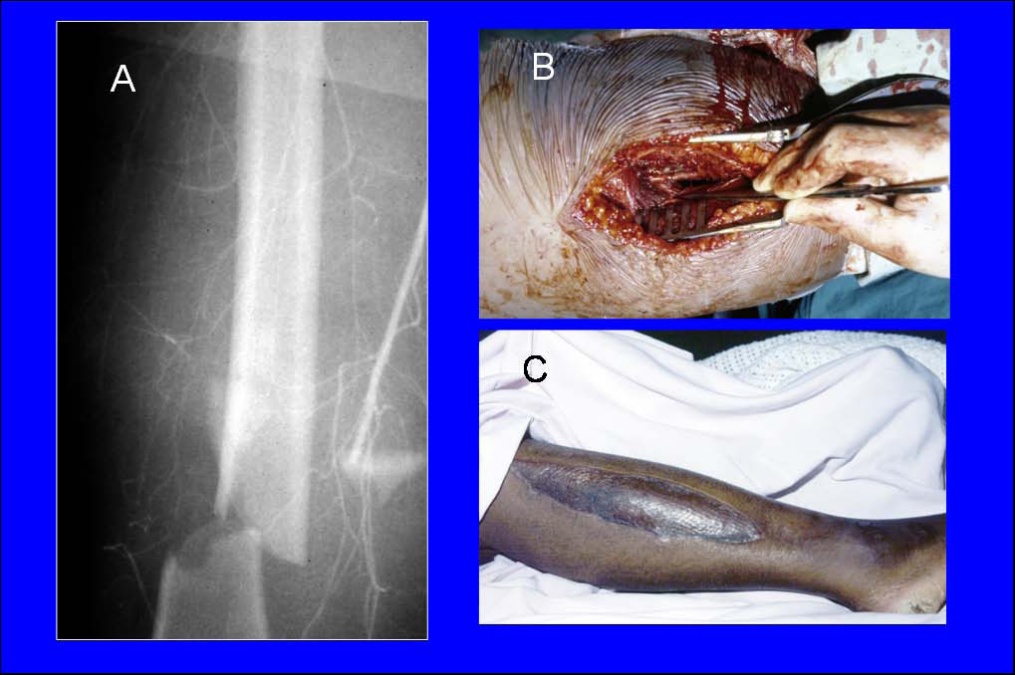
**A 24-years front seat female passenger who sustained fracture right femur with femoral artery injury (A) that needed venous interposition graft (B) and a fasciotomy of the right leg (C)**.

**Figure 4:**
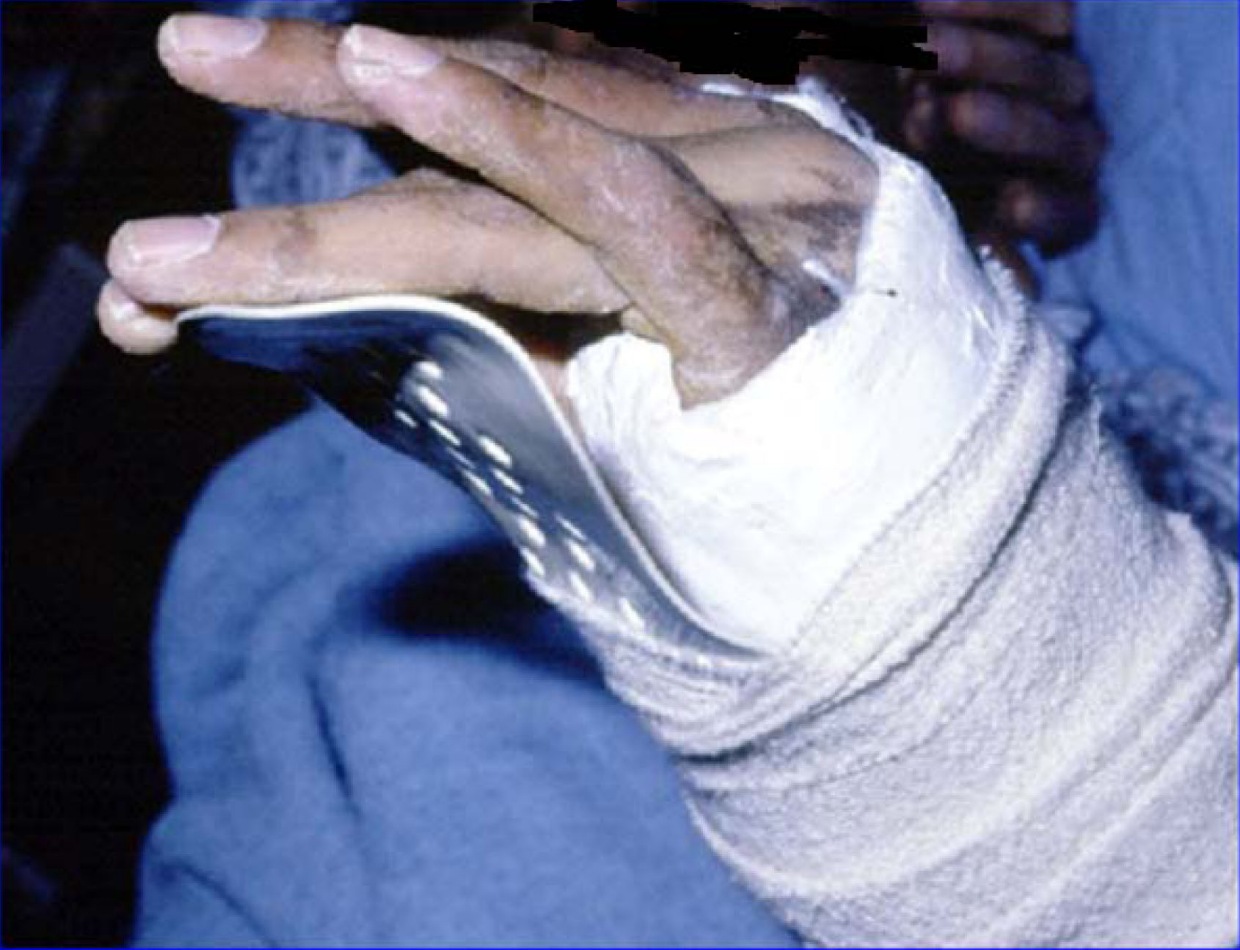
**A 25-years old laborer who had radial neck fracture and drop wrist**.

A two hour problem-solving, interactive tutorial on traumatology was structured to cover main topics in trauma management. The tutorial was based on real cases that demonstrated core learning objectives. The first author (FAZ) was personally involved in the management of these cases. The tutorial was built up to be standardized in a semi-controlled situation. All tutorials were done by the same tutor (FAZ) who had developed the educational material, covering the same cases, in the same format, sequence, and structure, and having specific objectives (Table 1). Figures 1, 2, 3 and 4 demonstrate some of these cases. Slide projectors were used without animation. The tutorial was structured to show a visual aid (slide), ask the question, define the problem, let students enquire and debate; even sometime in small groups, before a solution is reached. Slides were prepared according to scientific advised standards [6,7].

**Table 1:** Structure and objectives of the interactive problem-solving trauma tutorial

Case	Clinical hsitory	Questions asked	Objectives of the case
**1**	A 58-years old male fell on his left heel from 15 meters high.	What are the possible injuries of this patient?	Understand the biomechanics of blunt trauma; anticipate injuries depending on mechanism including pelvis, spine and abdominal organs.

**2**	A 20-years old male shot by a high energy bullet at right side of chest with an exist in the left loin **(Fig **1).	What are the possible injuries of this patient and how would you manage him?	Understand the biomechanics of ballistic injuries, draw the track of the bullet, appreciate the devastating severity of injury, and understand the need to stop bleeding and contamination.

**3**	30-years old front seat passenger with severe wind screen facial injury.	What do you think has happened? What are your priorities in management?	Understand the biomechanics of deceleration injuries of road traffic collisions, the importance of seatbelts and the need for airway protection.

4	A 30-years old soldier had a penetrating missile injury to his left chest and presented in shock.	What is shock and how can we find its cause?	To differentiate between different causes of shock (hypovolemia due to thoraco-abdominal injury, tension pneumothorax or pericardial tamponade), be able to systematically read a trauma chest X-ray.

5	45-years old male having a chest tube who developed severe hypoxia while being on ventilation **(Fig **2).	What are the possible reasons for hypoxia in this patient?	Understand causes of hypoxia in ventilated patients; stress the importance of logical analytical thinking to be able to solve this difficult problem.

6	An 18-years old male involved with a quarrel, hit on the left side of the head, in coma.	Can you read this brain CT scan (extradural haematoma)	Be able to identify acute intracranial bleeding, differentiate between extradural, subdural and intra-cerebral bleeding, and correlate the injury with neuroanatomy.

7	A 27-years old male involved with a car accident, has coma and pin point pupils, normal CT scan of the brain.	Why is the patient in coma? Where is the injury?	Appreciate the need to manage the patient and not the CT scan, limitations of trauma brain CT scan, importance of neurological examination to diagnose brain stem lesions.

8	A 24-years front seat female passenger involved in a car accident complaining of severe pain and deformity of the right thigh **(Fig **3).	What is the cause of pain in this patient? How can she be managed?	Appreciate the need to control pain in the trauma patients and know its cause, evaluate an extremity for neurovascular injury, appreciate the value for fasciotomy.

9	25-years old laborer fell from 3 meters high on his left forearm, had radial neck fracture and drop wrist **(Fig **4).	What nerve is injured?	Discuss the nerve distribution of the hand and clinical presentation of different nerve injuries; understand the importance of function recovery and rehabilitation in trauma management.

10	A 19-years old male who had a fracture femur treated with skeletal traction for three days develops sudden dyspnea?	What is the cause of his dyspnea, and how can we manage it?	Differentiate between ARDS and pulmonary embolism, pathophysiology, diagnosis and management.

### Room setting and procedures

**Figure 5:**
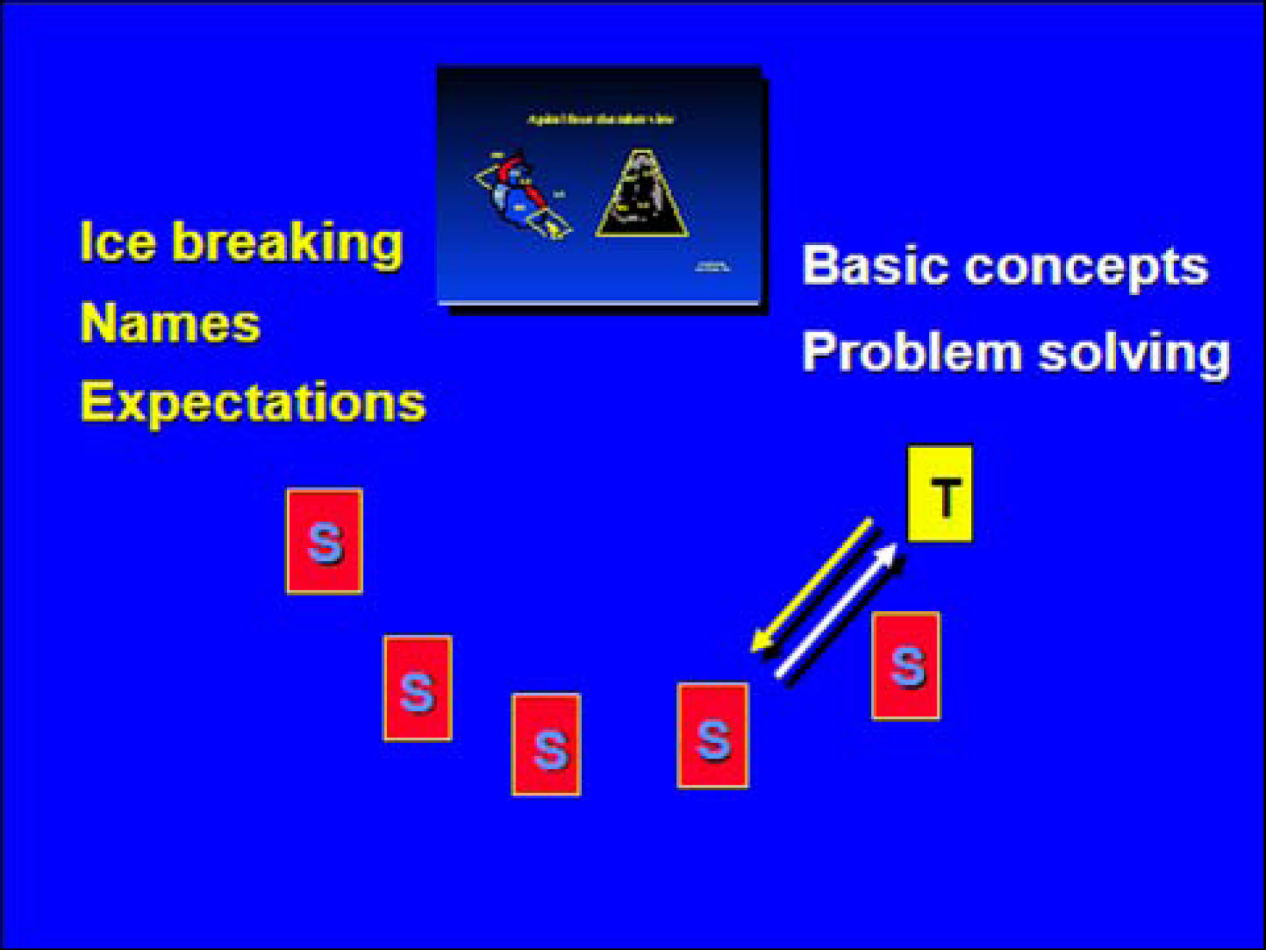
**A diagrammatic scheme showing the room setting**. The tutor (T) facilitates the interactive session by prompting the students (S) to think by asking questions leading to understand basic principles of trauma management.

Figure 5 demonstrates the room setting. The tutor stood on the side of the room so that the cases become the core of interest and not the tutor. Cards with names of the students were prepared in advance and put on their desk to help remembering their names. Ice breaking started by asking the students to present their names and what they expected from the tutorial. Ground rules were simple which included 1) everyone should participate, 2) explain why do you have this opinion 3) do not interrupt when others speak, 4) you can disagree but give an argument for that, 5) ask if things are not clear for you.

### Subjects

Seven tutorials, having 5-9 students each, were given to fourth year medical students at the Faculty of Medicine, Auckland, New Zealand (3 tutorials) and subsequently to fifth year students at the Faculty of Medicine, Al Ain, United Arab Emirates (4 tutorials) during the period of 1997-2001. Students were exposed to the tutor for the first time, had limited knowledge of trauma and had been used to a traditional, didactic approach to teaching and learning medicine. Significant student participation was expected and encouraged. A total of 50 students have attended these tutorials.

At the end of tutorial sessions, a reproduced self-administered questionnaire was utilized to gain students' feedback. This questionnaire consisted of 16 validated items focusing on the educational tool, tutor-based skills, and student-centered skills (Table 2). These items were selected from the Student Evaluations of Courses and Teaching booklet, Centre for Professional Development, Auckland University [8]. The advised number of items to be selected was 9 to 19 depending on what is needed to be evaluated. Areas selected were attitude with students, audiovisual aids, communication skills, motivation, and organization. Students anonymously rated items on a 7 point Likert-type scale. 15 items had the scale of (1 = very poor, 2 = poor, 3 = mediocre, 4 = acceptable, 5 = good, 6 = very good, and 7 = outstanding). Only one attribute (pace of presentation) was different (1 = too slow, 4 = just right, 7 = much too fast). Space was also provided for open-ended comments to the question "what did you like most about this person's lecturing?"

**Table 2:** Mean (SD) and median (range)) values for students' responses regarding the interactive approach to teaching traumatology (n = 50)

Attribute	Mean (SD)	Median (range)
**Educational tool**		
Use of real world cases	6.36 (0.75)	7 (5-7)
Use of visual methods	6.32 (0.62)	6 (5-7)
**Tutor-centred skills**		
Instructors enthusiasm for the subject	6.22 (0.7)	6 (5-7)
Ability to present the material in an interesting manner	6.06 (0.77)	6 (4-7)
Knowledge of the subject	5.94 (0.79)	6 (5-7)
Clarity of speech	5.92 (1)	6 (3-7)
Ability to structure the lecture in a clear manner	5.9 (0.81)	6 (4-7)
Ability to hold student's attention	5.8 (0.86)	6 (3-7)
Explains the material clearly	5.78 (0.98)	6 (3-7)
Pace of presentation (1 = too slow, 4 = just right, 7 = much too fast)	4.28 (0.67)	4 (4-7)
**Student-centered skills**		
Opportunity for students to ask questions	5.72 (1)	6 (3-7)
Amount learned overall (1 = nothing/7 = a lot)	5.72 (0.95)	6 (4-7)
Mix of theory and practice	5.64 (1.16)	6 (1-7)
Response to questions in a constructive way	5.59 (0.99)	6 (3-7)
Usefulness of class discussions	5.56 (1)	6 (3-7)

Overall effectiveness of teaching	5.98 (0.75)	6 (4-7)

### Statistical analysis

Students' feedback data were coded and entered into IBM compatible computers using the software program. The mean value of 14 out of 16 attributes was calculated for each student. This mean had a normal distribution. The variation of the means of different tutorials was homogenous (p = 0.78, Leven test). Two attributes were excluded from the calculation of the mean of attributes (the overall effectiveness of teaching and the pace of presentation because the best value was 4 and not 7 in this attribute). Data were analyzed with the PASW Statistics version 18, SPSS Inc, Chicago, Illinois, USA. The Cronbach's Alpha coefficient was calculated as a test of the internal consistency of the survey instrument. One way ANOVA analysis or Kruskall-Wallis as appropriate was used to test for difference between the 7 tutorials. Spearman rank correlation test was used to correlate the mean of attributes with the overall effectiveness of teaching. A p value of ≤ 0.05 was considered significant.

Students' open-ended comments were analysed qualitatively to explore the content of commentaries, perceived teaching strengths and weaknesses and attitudes to the interactive lecture approach.

## Results

All students at both universities returned completed questionnaires (100% response). The questionnaire had good internal validity having a Cronbach's Alpha of 0.87.

Table 2 shows the values for students' responses regarding the interactive approach including the educational tool, tutor-centered skills, and student-centered skills. It is clear that the educational tools were ranked higher. The median rank of the real world cases was outstanding followed by the use of slides. It is also evident that the mean tutor-centered skills were higher than the student-centered skills. The lowest ratings were for "response to questions in a constructive way" and "usefulness of class discussions".

**Figure 6:**
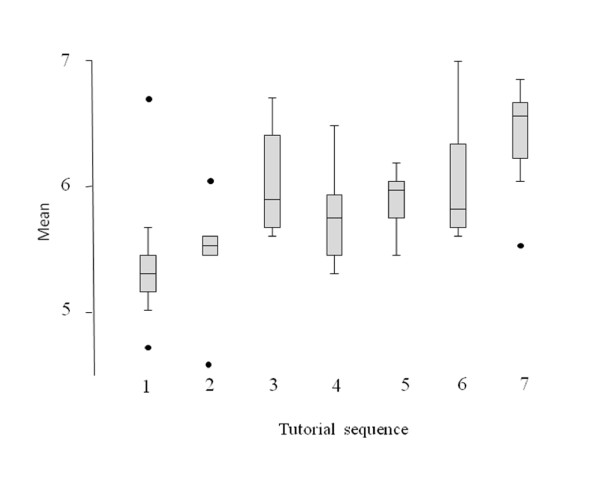
**Box plot of the mean of ratings of the attributes of the questionnaire**.

There was a significant correlation between the mean of attributes with the overall effectiveness of teaching (p < 0001, rho = 0.78, Spearman rank correlation). Figure 6 shows the mean of attributes in the 7 tutorials over time. There was a significant increase of the mean of attributes over time (F = 3.9, p = 0.004, ANOVA). There was also a very strong trend for improvement in the overall effectiveness of teaching (p = 0.058, Kruskall Wallis test).

Sixteen Al-Ain and 14 Auckland students offered open-ended comments (60%). All comments were supportive of use of the interactive lecture approach, practical examples, enthusiasm and clarity of the instructor. Typical comments are presented in Table 3 from which slight differences in length and fluency of comments are discernible.

**Table 3:** What did you like best about this tutor's teaching? Typical student comments

Comments Al-Ain students	Comments Auckland students
The kind of lecturing which depends on student discussion and questioning which can hold the attention of the students for maximal time	It was interesting. The tutor was enthusiastic and that made me enthusiastic. He had a good approach because rather than lecturing to us he got us to participate. I liked the way he choose particular students to answer questions as some students are quieter and would like to answer questions but often do not come forward quickly - he made it so these students got the opportunity to come forward

Introduction, slide presentation; group discussion and brain storming; starting from how much we understood and then adding to it	Nice slides; enjoyed the introduction

"Ice-breaking", clear illustrations; explanations of all facts presented	Portrayed his immense knowledge really well; very interesting and his enthusiasm is infective

Way of discussion; asking students questions, using real and good cases	His topic; the way he asked questions to individuals and was open to questions. Relaxed environment; talked with us, not at us

Giving practical and real examples	Good use of slides and photos relevant to real world. Explanations clear; opportunity for questions good; interesting material presented in a clear manner.

Use of real life slide; encouraging us to participate and understand the material by asking and answering questions; not only lecturing	Variety of examples given was great; incorporation of theory into slide presentations; management scheme given, not just advice on parts of management

Beautiful examples matching with reality	Good use of practical examples - how trauma occurred, what that means and what to do

## Discussion

Competition on the curriculum space, the need for student-centered learning, and a direction towards more medical care in the community, have reduced the time for teaching undergraduate surgery. Obligatory surgical rotations of the undergraduate curriculum have declined by almost 30% in the United States [9]. We have realized over time the need to promote problem-oriented, [10] patient-centered [11], and student-centered [12] approaches in surgical education of medical students. We have, at the same time, the challenge to expose students to multiple surgical problems to be solved. This is very relevant to an area of wide diversity like trauma in which respecting well defined rules are essential for a better patients' outcome [13]. Nevertheless, using analytical deductive methods are the safe guard when unusual cases are faced [14,15]. It is a challenge to develop the students' thinking at an early stage parallel with their knowledge.

The tutorial which was developed has an advantage of exposing the students to different problems of varying difficulties within a short time. The simple problem can be solved easily using the pattern diagnosis, like the case of radial nerve injury (case 9, Table 1). More difficult cases, like developing a tension pneumothorax despite a chest tube, and a serious brain stem lesion despite a normal CT scan (cases 5 and 7, Table 1), need more deeper thinking, and understanding of the basic sciences to be solved [14,15].

There is an increasing trend toward actively involving students in their learning. Several authors support the view that active, experiential learning contribute to perceived student satisfaction with teaching [16,17]. These methods engender greater cognitive engagement, more student-student and student-instructor interaction. Perceptions of learning activities cannot be predicted in advance. Therefore it cannot be assumed that learners will achieve the aim of an activity as intended by course designers and instructors [18]. So it is essential to evaluate different educational activities regularly.

On the whole, students both in Auckland and Al-Ain considered the interactive lecture on the topic of traumatology very effective. Students' perceptions regarding the relative importance of specific tutor behaviors was ranked less than the interactive approach itself. Nevertheless, the tutor-centered instructional skills were ranked higher than the student-centered learning skills. We have before found that student-centered instructional skills need to be improved [12]. The first author (FAZ) tried to modify his teaching methods accordingly. Nevertheless, the present study highlights that he still needs to work more on this area. An earlier study conducted in the UAE University, Faculty of Medicine indicated that characteristics identified as most important by students and Faculty included ability for clear communication in simple language, ability to present information in a logical sequence, and to create an atmosphere for discussion [19]. Response to questions in a constructive way and usefulness of class discussions had relatively the lowest rank in the present study although their rating was high having a median rank of 6 out of 7.

Students' comments revealed that both groups valued highly the interactive approach to teaching and learning and open-ended comments indicate that they appreciated instructor questioning, encouragement of active involvement and participation. Despite that, these were ranked less than the tutor-centered instructional skills.

Studies of interactive lectures in various disciplines, including medicine, are stimulating, promote student and teacher satisfaction, engagement, and motivation [3,20,21]. Nevertheless, as Steinert and Snell [3] indicate interactive approaches require utilization of various forms of questioning which "can stimulate interest, arouse attention, serve as an 'ice-breaker' and provide valuable feedback to the teacher and student alike". Questioning and probing students effectively are skills that educators should be trained on during teaching enhancement programs for Faculty [22,23].

The dynamics of the tutorial process is multifaceted including the educational methods, the tutor, and the learners. Concentrating on one of them will lead to an incomplete understanding of the educational process [24]. Thus, it is important to take a holistic approach to evaluate teaching and learning. This opinion was supported by others [25]. Contemporary instructional strategies that considers only instructor behaviors, is unlikely to succeed in improving the quality of education. Action should be done at the same time on educational methods and promoting active students' learning. We tried to achieve that by developing an educational tool which actively involves the students in the learning process.

### In summary

The interactive problem-solving approach for tutorials can be an effective enjoyable alternative or supplement to traditional instruction for teaching traumatology to medical students. Training for this approach should be encouraged for Faculty development.

## Consent

An informed consent was taken from patients to use their images for medical education/publication.

## Competing interests

The authors declare that they have no competing interests.

## Authors' contributions

FAZ had the idea, designed the study, collected and analyzed the data, wrote the manuscript, repeatedly edited it, and approved its final version. MAE helped in the idea, analysis of the data, writing of the manuscript, and approved the final version of the paper.
